# Excessive weight gain onset-age and risk of developing diabetes mellitus: a large, prospective Chinese cohort study

**DOI:** 10.3389/fendo.2023.1281203

**Published:** 2023-11-27

**Authors:** Wei Fang, Xiaojie Yuan, Weijian Li, Samuel Seery, Guanzhi Chen, Zefeng Cai, Zegui Huang, Xianxuan Wang, Weiqiang Wu, Zhichao Chen, Yan Li, Shouling Wu, Youren Chen

**Affiliations:** ^1^ Department of Cardiology, Second Affiliated Hospital of Fourth Military Medical University, Xi’an, China; ^2^ Department of Epidemiology, School of Public Health, Fourth Military Medical University, Xi’an, China; ^3^ Shantou University Medical College, Shantou, China; ^4^ Department of Cardiology, Second Affiliated Hospital of Shantou University Medical College, Shantou, China; ^5^ Faculty of Health and Medicine, Division of Health Research, Lancaster University, Lancaster, United Kingdom; ^6^ China Medical University, Shenyang, China; ^7^ Department of Cardiology, Kailuan General Hospital, Tangshan, China

**Keywords:** overweight, obesity, onset age, diabetes mellitus, prospective cohort

## Abstract

**Background:**

Excessive weight gain and obesity are widely accepted as risk factors for diabetes mellitus, and the age at which obesity onsets may be related to the development of cardiovascular diseases and certain cancers. Here, we aimed to investigate associations between the onset-age of overweight/obesity and risk of developing diabetes mellitus in China.

**Methods:**

42,144 people with the normal weight range and without diabetes at baseline, were enrolled from the Kailuan cohort which began on the 1^st^ June 2006. All participants were followed-up, biennially, until 31^st^ December 2017. During follow-up, 11,220 participants had become overweight/obese. For each case, one normal-weight control was matched according to age ( ± 1 year) and sex. Our final analysis included 10,858 case-control pairs. An age-scaled Cox model was implemented to estimate hazard ratios (HR) with corresponding 95% confidence intervals (CI) for diabetes mellitus incidence across age-groups.

**Results:**

At a median follow-up of 5.46 years, 1,403 cases of diabetes mellitus were identified. After multivariate adjustments, age-scaled Cox modelling suggested that risk gradually attenuated with every 10 year increase in age of onset of overweight/obesity. Diabetes mellitus adjusted HRs (aHRs) for new-onset overweight/obesity at <45years, 45-54 years, and 55-64 years were 1.47 (95%CI, 1.12-1.93), 1.38 (95%CI, 1.13-1.68), 1.32 (95%CI, 1.09-1.59), respectively. However, new-onset of overweight/obesity at ≥65 years did not relate to diabetes mellitus (aHR, 1.20; 95%CI, 0.92-1.57). This trend was not observed in women or the new-onset obesity subgroup but was evident in men and the new overweight onset subgroup.

**Conclusion:**

Participants with early onset of excessive weight gain issues are at considerably higher risk of developing diabetes mellitus compared to those who maintain a normal weight.

## 1 Introduction

Lifestyles change substantially with increased economic prosperity although these changes are not always positive. We are witnessing a type 2 diabetes mellitus pandemic which is closely linked to sedentary lifestyles and weight-gain; however, the prevalence of type 1 is also rising. Diabetes has therefore become a major public health concern in both India and China, where there has been substantial economic development and almost one 5^th^ of the world’s population reside. In China alone, the prevalence of overweight/obese adults is approximately 34.3% and 16.4%, respectively (1, 2). Perhaps even more alarming is the prevalence of obesity in children which is rapidly rising (3, 4).

Coincidentally, the prevalence of diabetes has rapidly increased from 9.7% in 2007 to 11.2% in 2017 among Chinese adults (5). Therefore, special attention must be paid to excessive weight gain and obese individuals in China because diabetes mellitus creates a huge economic burden for governments and for individuals, who not only encounter well-known macro and micro-vascular complications, but also encounter depression, anxiety, and all too frequently, die early (6, 7). In more developed societies, excessive weight gain and obesity are widely accepted as risk factors for diabetes mellitus. However, there are genetic differences and lifestyle factors which contribute to insulin resistance and therefore the prevalence of diabetes varies between nationalities and within ethnicities (8). Chinese researchers have postulated that multisectoral efforts are required to address the diabetes epidemic in China; however, these efforts must not be entirely reactive. We need to develop evidence-based preventive strategies to tackle this growing problem.

Demarcation between pre-symptomatic diabetic cases and those who encounter symptoms remains unclear, especially for the public. For example, people may attribute fatigue and macro and micro-vascular issues to ageing rather than being signs of diabetes which should initiate health seeking behaviors. Given the magnitude of the clinical iceberg in China, this is not always the case and so we, as a global community must learn about the differences between and within nationalities in order to identify (and intervene) pre-symptomatic cases. Two studies from Kailuan cohort found that hypertension and diabetes mellitus are risk factors for developing cardiovascular diseases (CVD), which were different across different onset ages in China (9, 10). Further research has suggested that the age at which obesity onsets may be related to the development of cardiovascular diseases and certain cancers (11, 12). However, little is known about correlations between the age of onset of overweight/obesity and risk of developing diabetes mellitus, especially across the mainland Chinese population. It is also hoped that by studying a large, Chinese cohort, we will add to the comparative evidence-base to ensure public health interventions are more highly specified. Therefore, we aimed to investigate associations between the onset-age of overweight/obesity and risk of developing diabetes mellitus in China.

## 2 Methods

### 2.1 Study design and participants

This is an exposure-control matched cohort study based on Kailuan cohort in Tangshan, Hebei province. From June 2006 to October 2007, participants from the Kailuan community completed questionnaires and a first survey in Kailuan General Hospital and 10 affiliated hospitals. Subsequent surveys including questionnaires and blood tests were provided every two years, in 2008 to 2009, 2010 to 2011, 2012 to 2013, 2014 to 2015, and 2016 to 2017. All questionnaires were completed by trained nurses and blood tests were taken by laboratory technicians. Data entry was performed by double entry using Epidata, and those with more than half the required data missing were excluded.

A total of 101,510 participants aged between 18-98 years were recruited between 2006 to 2007. After excluding 59,366 participants who did not have baseline body mass index (BMI) or fasting plasma glucose (FBG) information (n = 313), overweight/obese participants (n = 42,586), 3,144 with low-weight, had been diagnosed with diabetes mellitus (n = 9,489) at baseline, or 3,834 lost to follow-up and there were 42,144 participants available for matching (**Figure S1A**). From 2008 to 2015, 11,220 were considered new-onset excessive weight gain, which included overweight cases and those considered obese. After randomly matching participants with overweight or obese participants with those who maintained a normal BMI across the follow-up period according to age (+/- 1 year), sex and visitations, a total of 21,716 participants (overweight/obese, n=10,858; normal-weight, n=10,858) were finally included. The study was based on The Kailuan Study (trial identification: ChiCTR-TNC-11001489), approved by ethics committee of Kailuan General Hospital. Informed consent was required before individuals were granted access to participate.

### 2.2 Exposure: new-onset weight gain, considered overweight/obesity

During each examination, weight and height were recorded using calibrated RGZ-120 scales with participants removed footwear and over-clothes. Measures were rounded to the nearest 0.1 kg for weight and 0.1 cm for height. Weight status was ascertained according to BMI, which was calculated by dividing body weight (kg) by height squared (m^2^). Cut-off points were predetermined according to the World Health Organization’s standard ranges (i.e. underweight, BMI < 18.5 kg/m^2^; normal-weight, 18.5 ≤ BMI <25.0 kg/m^2^; overweight, 25.0 ≤ BMI < 30.0 kg/m^2^; and obesity, BMI ≥ 30.0 kg/m^2^) (13).

New-onset weight gain was defined according to BMI changes from normal-weight at baseline to overweight or obese recorded prior to 31^st^ December 2015 or the point of diabetes mellitus diagnosis. Every case was matched with a control who maintained normal-weight across the follow-up period, according age (+/-1 year), sex and visit time (**Figure S1B**).

### 2.3 Outcomes: diabetes mellitus incidence during follow-up

Diabetes mellitus incidence was determined according to FBG ≥7.00 mmol/L (126 mg/dL) and/or treatment with anti-hyperglycemic drugs, and/or self-reported physician-diagnosed diabetes mellitus during the follow-up period according to American Diabetes Association guidelines (14). Blood samples from each participant were collected in the morning of each survey after at least a 12-hour fast. FBG was tested using hexokinase method by automatic biochemical analyzer (Hitachi 747; Hitachi, Tokyo, Japan). Diabetes histories and related treatments were collected by trained nurses through structured questionnaires (details are provided in the **Supplementary Materials**). We did not further distinguish type 1 or type 2 diabetes mellitus. The baseline for this study was defined according to the onset time of excessive weight gain or the time that participants with normal-weight were matched. All participants were followed-up until the date of diabetes mellitus diagnosis or until the final visit on the 31^st^ December 2017, whichever came first.

### 2.4 Assessment of covariates

Data around other related variables were also collected and updated through questionnaires and blood tests every two year. Covariates were derived from the examination year at which each matched pair was confirmed. Family history of diabetes, education level, physical activity, cigarette smoking and alcohol drinking status were obtained through self-reported questionnaires. Education level was defined as “less than high school”, “high school”, or “university degree or higher”. Active physical activity was defined as aerobic exercise ≥ 3 times per week. Smoking and alcohol drinking status were stratified into three levels: “current”, “former” and “never”. Total cholesterol (TC), triglyceride (TG), high-density lipoprotein-cholesterol (HDL-C) and low-density lipoprotein-cholesterol (LDL-C) were also tested using an automatic biochemical analyzer (Hitachi 747; Hitachi, Tokyo, Japan). Blood pressure was measured three times, and mean systolic blood pressure (SBP) and diastolic blood pressure (DBP) was used.

### 2.5 Statistical analysis

New-onset weight gain including participants with overweight or obesity and matched controls were stratified into four groups according to onset age: <45 years, 45-54 years, 55-64 years and ≥65 years. Continuous variables were expressed as means with corresponding standard deviations (SD) and compared using Student’s t test or one-way analysis of variance (ANOVA) analysis. Categorical variables were shown as proportions and compared using a standard Chi-Square test.

An age-scaled Cox regression model, which took age rather than follow-up time as the time scale, was used to calculate the hazard ratios (HR) and 95% confidence intervals (CI) for risk of developing diabetes mellitus at new-onset overweight/obesity, compared with normal-weight participants across age-groups (15). Multivariate adjusted models were implemented for systolic blood pressure (SBP), FBG, TG, HDL-C, LDL-C, cigarette smoking status, alcohol drinking status, physical activity, family history of diabetes and education level, considering a high collinearity between SBP and DBP as well as TC, HDL-C and LDL-C. A further form of subgroup analysis compared by sex and among overweight and obese participants, separately. To evaluate fluctuations in body weight, we conducted subgroup analysis of those who reduced their weight to within the normal BMI range and those who maintained their overweight or obese status.

To test robustness and address the potential for reverse causation, we further performed sensitivity analyses by excluding participants who were diagnosed with malignant tumors during study, and those who encountered diabetes mellitus within the first year of follow-up.

All analyses were performed using SAS (Version 9.4). A fully conditional specification method was used to impute missing values for covariates using multivariate imputation by chained equation (MICE) method (16, 17). Details of missing covariates were presented in **Table S1**. All statistical tests were two-sided, and p <0.05 set as the threshold for statistical significance.

## 3 Results

### 3.1 Baseline characteristics

A total of 21,716 participants (overweight/obese, n=10,858; normal-weight, n=10,858) were finally included. Aggregated participant characteristics are provided in **Tables 1**, **2**. Compared with participants categorized as normal-weight, new-onset overweight/obese subjects had higher FBG, SBP, DBP, TC, TG and LDL-C level, but lower HDL-C levels and a lower prevalence of smokers (**Table 1**). Among people with new-onset overweight/obesity a younger onset age correlated with lower FBG, SBP, DBP, TC, HDL-C, and LDL-C levels (**Table 2**). Additionally, these people were more likely to be smokers, alcohol drinkers, and physically inactive. They also had higher levels of TG and education overall but an increased prevalence in the family history of diabetes compared to those with an older onset age.

**Table 1:** Baseline characteristics of new-onset overweight/obesity and normal-weight controls†.

Variables	New-onset overweight/obesity	Normal-weight	*P*
Participants, n	10,858	10,858	
Age, years	52.71 ± 12.21	52.71 ± 12.21	-
Male sex, n(%)	8018(73.8)	8018(73.8)	-
BMI, kg/m^2^	26.52 ± 2.36	22.24 ± 1.55	<0.01
FBG, mmol/L	5.26 ± 0.62	5.17 ± 0.61	<0.01
SBP, mmHg	131.01 ± 19.13	126.37 ± 18.95	<0.01
DBP, mmHg	84.04 ± 10.70	81.19 ± 10.40	<0.01
TG, mmol/L	1.63 ± 1.27	1.33 ± 1.14	<0.01
TC, mmol/L	5.05 ± 0.99	4.97 ± 1.00	<0.01
HDL-C, mmol/L	1.46 ± 0.46	1.56 ± 0.47	<0.01
LDL-C, mmol/L	2.65 ± 0.88	2.56 ± 0.85	<0.01
Cigarette smoking, n(%)			
Current	3174(29.2)	3486(32.1)	<0.01
Former	840(7.8)	790(7.3)	
Never	6844(63.0)	6582(60.6)	
Alcohol drinking, n(%)			
Current	2235(20.5)	2287(21.1)	0.55
Former	1983(18.3)	1935(17.8)	
Never	6640(61.2)	6636(61.1)	
Physical exercise, n(%)	1841(17.0)	1851(17.0)	0.86
Family history of diabetes, n(%)	934(8.6)	926(8.5)	0.85
Education, n(%)			0.05
Less than high school	860(7.9)	824(7.6)	
High school degree	9165(84.4)	9108(83.9)	
University degree or higher	833(7.7)	926(8.5)	

BMI, body mass index; DBP, diastolic blood pressure; FBG, fasting blood glucose; HDL-C, high-density lipoprotein-cholesterol; LDL-C, low-density lipoprotein-cholesterol; SBP, systolic blood pressure; TC, total cholesterol; TG, triglycerides.

† Baseline refers to the examination cycle when new-onset overweight/obesity was first identified.

**Table 2:** Baseline characteristics of new-onset overweight/obesity across age groups†.

Variables	New-onset overweight/obesity according to age				*P*
	<45 years	45-54 years	55-64 years	≥65 years	
Participants, n	2,739	3,284	3,258	1,577	
Age, years	36.8 ± 5.9	50.2 ± 2.9	59.4 ± 2.8	71.7 ± 5.3	–
Male sex, n(%)	2049(74.8)	2215(67.4)	2439(74.9)	1315(83.4)	–
BMI, kg/m^2^	26.5 ± 2.3	26.5 ± 2.5	26.5 ± 2.2	26.5 ± 2.4	<0.01
FBG, mmol/L	5.1 ± 0.6	5.2 ± 0.6	5.3 ± 06	5.4 ± 0.6	<0.01
SBP, mmHg	121.7 ± 14.7	128.8 ± 17.3	136.0 ± 19.7	141.6 ± 20.0	<0.01
DBP, mmHg	81.2 ± 9.9	84.5 ± 10.8	85.8 ± 10.9	84.5 ± 10.6	<0.01
TG, mmol/L	1.8 ± 1.4	1.7 ± 1.4	1.6 ± 1.1	1.4 ± 0.9	<0.01
TC, mmol/L	4.9 ± 1.0	5.1 ± 1.0	5.2 ± 1.0	5.1 ± 1.0	<0.01
HDL-C, mmol/L	1.4 ± 0.4	1.5 ± 0.5	1.5 ± 0.5	1.5 ± 0.5	<0.01
LDL-C, mmol/L	2.6 ± 0.8	2.6 ± 0.9	2.7 ± 0.9	2.7 ± 1.0	<0.01
Cigarette smoking, n(%)					<0.01
Current	1001(36.6)	1101(33.5)	803(24.7)	269(17.1)	
Former	184(6.7)	226(6.9)	294(9.0)	136(8.6)	
Never	1554(56.7)	1957(59.6)	2161(66.3)	1172(74.3)	
Alcohol drinking, n(%)					<0.01
Current	744(27.2)	746(22.7)	522(16.0)	223(14.1)	
Former	624(22.8)	662(20.2)	500(15.4)	197(12.5)	
Never	1371(50.0)	1876(57.1)	2236(68.6)	1157(73.4)	
Physical exercise, n(%)	257(9.4)	467(14.2)	719(22.1)	398(25.2)	<0.01
Family history of diabetes, n(%)	373(13.6)	345(10.5)	189(5.8)	27(1.7)	<0.01
Education, n(%)					<0.01
Less than high school	39(1.4)	115(3.5)	333(10.2)	364(23.1)	
High school degree	2163(79.0)	3014(91.8)	2840(87.2)	1155(73.2)	
University degree or higher	537(19.6)	155(4.7)	85(2.6)	58(3.7)	

BMI, body mass index; DBP, diastolic blood pressure; FBG, fasting blood glucose; HDL-C, high-density lipoprotein-cholesterol; LDL-C, low-density lipoprotein-cholesterol; SBP, systolic blood pressure.

† Baseline refers to the examination cycle when new-onset overweight/obesity was first identified.

### 3.2 Diabetes mellitus incidence

**Figure 1:**
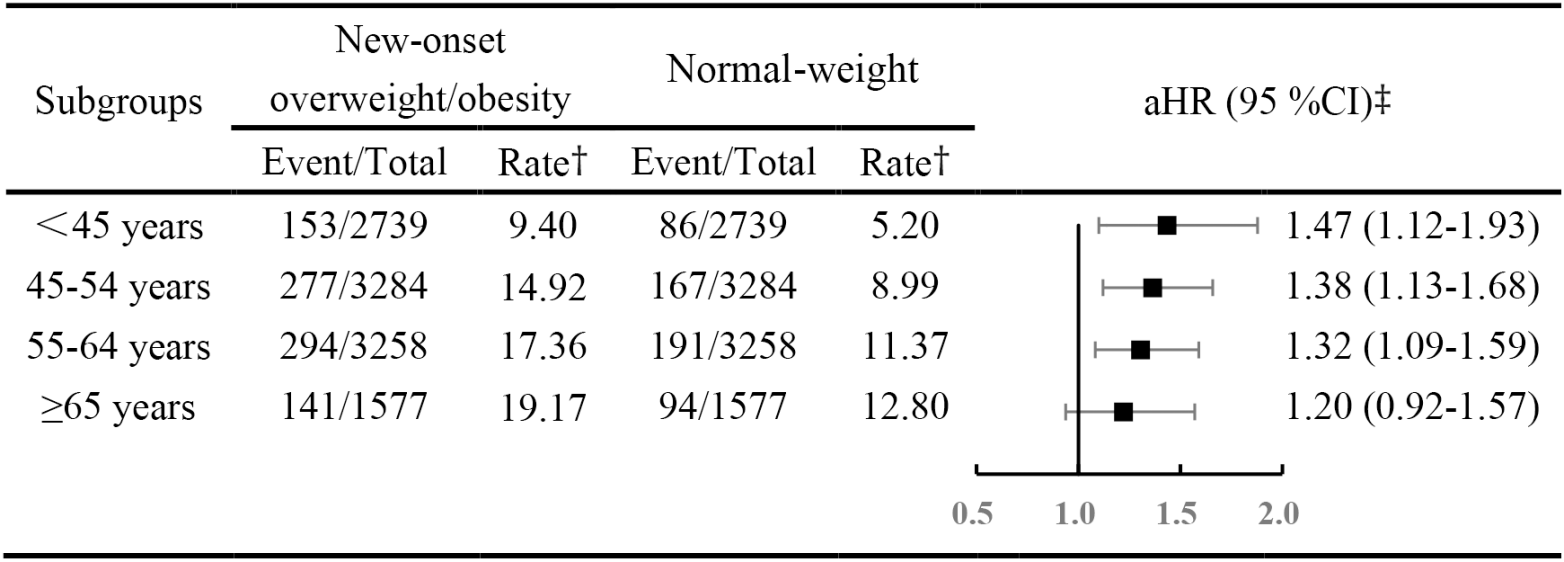
Hazard rations for diabetes mellitus across age-based onset groups, among new onset overweight and obesity versus normal-weight participants. † The rate was per 1,000 person years. ‡ Adjusted for systolic blood pressure, fasting blood glucose, triglyceride, high-density lipoprotein-cholesterol, low-density lipoprotein-cholesterol, cigarette smoking status, alcohol drinking status, physical exercise, family history of diabetes, education degree. Abbreviations: aHR, adjusted hazard ratio; CIs, confidence intervals.

During a median of 5.46 years (118,381 person-years) follow-up, we identified 1,403 previously undiagnosed diabetes cases. Compared to those who maintained a normal-weight during follow-up, people with new-onset overweight/obesity showed a higher risk of developing diabetes (adjusted HR [aHR], 1.29; 95% CI 1.02-1.63). However, the risk was different across onset ages (*P* for interaction < 0.05). As shown in **Figure 1**, the incidence and rate of diabetes were higher in people with new-onset overweight/obesity across all age-groups. In conjunction with increasing age, the number and rate appeared to consistently increase.

Compared with those considered to have maintained a normal weight, people with overweight/obesity were at a higher risk of developing diabetes, after adjusting for education level, cigarette smoking and alcohol drinking status, physical activity, family history of diabetes, SBP, TG, LDL-C and HDL-C level. The risk of developing diabetes gradually attenuated with every decade increase in age at onset of overweight/obesity, with a aHR of 1.47 (95% CI, 1.12-1.93) in those onset age <45 years, 1.38 (95% CI, 1.13-1.68) in those onset age from 45 to 54 years and 1.32 (95% CI, 1.09-1.59) in those onset age from 55 to 64 years. Although, those whose onset age was 65 years or older did not appear at higher risk (aHR,1.22; 95% CI, 0.94-1.59).

### 3.3 Subgroup analysis

Stratified by sex, results did not change substantially in males, with an aHR of 1.56 (95% CI, 1.16-2.10) in those onset age<45 years, 1.31 (95% CI, 1.04-1.65) in those onset age from 45 to 54 years and 1.30 (95% CI, 1.05-1.62) in those onset age from 55 to 64 years (**Table 3**). However, a positive correlation was observed in the relationship between women with overweight/obesity whose onset age was between 45 to 54 years (aHR, 1.58; 95% CI, 1.09-2.28). Although this was not considered a significant interaction (*P* = 0.743).

**Table 3:** Hazard ratios for diabetes mellitus across age-based onset groups, among new-onset overweight and obesity versus normal-weight participants by sex.

Subgroup	New-onset overweight/obesity		Normal-weight		aHR (95% CI) †	*P* for interaction
	Event/Total	Rate†	Event/Total	Rate†		
Male (n=16,036)						0.74
<45 years	130/2049	10.63	73/2049	5.80	1.56 (1.16-2.10)	
45-54 years	185/2215	14.89	122/2215	9.80	1.31 (1.04-1.65)	
55-64 years	221/2439	17.49	145/2439	11.70	1.30 (1.05-1.62)	
≥65 years	116/1315	19.14	76/1315	12.59	1.21 (0.90-1.63)	
Female (n=5,680)						
<45 years	23/690	5.69	13/690	3.31	0.98 (0.47-2.04)	
45-54 years	92/1069	14.99	45/1069	7.32	1.58 (1.09-2.28)	
55-64 years	73/819	16.98	46/819	10.44	1.38 (0.94-2.03)	
≥65 years	25/262	19.35	18/262	13.77	1.27 (0.67-2.42)	

aHR, adjusted hazard ratio; CI, confidence interval.

† Adjusted for systolic blood pressure, fasting blood glucose, triglyceride, high-density lipoprotein-cholesterol, low-density lipoprotein-cholesterol, cigarette smoking status, alcohol drinking status, physical exercise, family history of diabetes, education degree.

We further divided the overweight/obesity group into participants with overweight and participants with obesity in order to compare risks of developing diabetes (**Table 4**). The overweight group had a significant association with DM occurrence, with an aHR of 1.44 (95% CI, 1.09-1.91) in those onset age<45 years, 1.37 (95% CI, 1.12-1.67) in those onset age from 45 to 54 years and 1.32 (95% CI, 1.09-1.59) in those onset age from 55 to 64 years. However, there was no significant finding in those considered obesity (All *P* > 0.05).

**Table 4:** Hazard ratios for incident diabetes mellitus among patients with new-onset overweight and obesity versus normal-weight participants, across separate age-groups.

Subgroup	New-onset overweight or obesity		Normal-weight		aHR (95% CI)‡
	Event/Total	Rate†	Event/Total	Rate†	
Overweight (n=20,692)					
<45 years	146/2623	9.35	84/2623	5.29	1.44 (1.09-1.91)
45-54 years	263/3113	14.90	161/3113	9.13	1.37 (1.12-1.67)
55-64 years	282/3125	17.30	184/3125	11.38	1.32 (1.09-1.59)
≥65 years	131/1485	18.76	88/1485	12.61	1.17 (0.89-1.54)
Obesity (n=1,024)					
<45 years	7/116	10.53	2/116	3.02	3.08 (0.25-37.89)
45-54 years	14/171	15.21	6/171	6.39	1.41 (0.53-3.78)
55-64 years	12/133	18.74	7/133	11.18	1.38 (0.53-3.58)
≥65 years	10/92	26.80	6/92	16.40	2.42 (0.58-9.98)

aHR, adjusted hazard ratio; CI, confidence interval.

† Rate was per 1,000 person years.

‡ Adjusted for systolic blood pressure, fasting blood glucose, triglyceride, high-density lipoprotein-cholesterol, low-density lipoprotein-cholesterol, cigarette smoking status, alcohol drinking status, physical exercise, family history of diabetes, education degree.

To assess the influence of weight fluctuations, we further divided the overweight/obese group into participants who were initially categorized as overweight or obese but who subsequently achieved normal BMI, and those who remained in the overweight or obese category (**Table 5**). Results showed that regardless of the onset age reducing weight to within a normal BMI range significantly reduced the risk of developing diabetes. The HR was 0.35 (95% CI, 0.24-0.49) in those with an onset age <45 years, and 0.31 (95% CI, 0.24-0.41) in those onset age from 45 to 54 years, 0.35 (95% CI, 0.27-0.45) in those onset age from 55 to 64 years and 0.30 (95% CI, 0.21-0.44) in those onset age ≥65 years.

**Table 5:** Hazard ratios for incident diabetes mellitus of changed to a normal BMI among patients with new-onset overweight and obesity, across separate age-groups.

Subgroup	Changed to a normal BMI		Stable overweight/obesity		aHR (95% CI)‡
	Event/Total	Rate†	Event/Total	Rate†	
<45 years	45/1264	5.45	108/1475	13.47	0.35 (0.25-0.50)
45-54 years	76/1659	7.51	201/1625	23.82	0.32 (0.25-0.42)
55-64 years	84/1533	9.33	210/1725	26.47	0.35 (0.27-0.46)
≥65 years	44/850	9.81	97/727	33.78	0.30 (0.21-0.43)

aHR, adjusted hazard ratio; CI, confidence interval.

† Rate was per 1,000 person years.

‡ Adjusted for systolic blood pressure, fasting blood glucose, triglyceride, high-density lipoprotein-cholesterol, low-density lipoprotein-cholesterol, cigarette smoking status, alcohol drinking status, physical exercise, family history of diabetes, education degree.

### 3.4 Sensitivity analysis

Results remained consistent after excluding participants who were diagnosed with malignant tumors during study and after excluding those with outcome events within the first year of follow-up (**Table 6**).

**Table 6:** Sensitivity analysis.

Subgroup	New-onset overweight/obesity		Normal-weight		aHR (95% CI) ‡
	Event/Total	Rate†	Event/Total	Rate†	
Excluding participants with malignant tumors (n=21,436)					
<45 years	153/2725	9.45	86/2725	5.37	1.37 (1.05-1.80)
45-54 years	273/3240	14.91	167/3240	9.41	1.31 (1.08-1.60)
55-64 years	291/3207	17.49	191/3207	11.54	1.26 (1.05-1.52)
≥65 years	138/1546	19.14	86/1546	11.90	1.27 (0.96-1.67)
Excluding participants with outcome events within the first year of follow-up (n=21,682)					
<45 years	153/2736	9.40	86/2736	5.2	1.45 (1.11-1.91)
45-54 years	275/3278	14.82	167/3278	8.99	1.35 (1.11-1.64)
55-64 years	294/3253	17.36	190/3253	11.31	1.29 (1.07-1.55)
≥65 years	140/1574	19.04	94/1574	12.8	1.21 (0.93-1.59)

aHR, adjusted hazard ratio; CI, confidence interval.

† Rate was per 1,000 person years.

‡ Adjusted for systolic blood pressure, fasting blood glucose, triglyceride, high-density lipoprotein-cholesterol, low-density lipoprotein-cholesterol, cigarette smoking status, alcohol drinking status, physical exercise, family history of diabetes, education degree.

## 4 Discussion

Our findings suggest that new-onset overweight/obesity is associated with a higher risk developing diabetes mellitus among Chinese mainlanders, although the magnitude of this effect varied across the lifespan. Participants considered (using BMI) as overweight or obese at age <45 years were at the highest risk of developing diabetes mellitus, compared with age- and sex- matched controls. The aforementioned risk gradually attenuated with each decade increase in excessive weight gain onset age. The results also remained stable in men and when analysis was constricted to overweight participants, but not for those considered obese.

To date, few studies have investigated the relationship between excessive weight gain onset age and risk of developing diabetes. In a related, British birth cohort study, which compared those who had never been obese, childhood obesity and younger-adulthood obesity have a 4.38-fold (95% CI, 1.86–10.31) and 3.96-fold (95%CI, 2.10-7.43) risk of hemoglobin A1c (HbA1c) ≥7%, respectively (18). HbA1c is an indicator for glucose metabolism, where a threshold of 6.5% could also be used to diagnose diabetes mellitus (19). An HbA1c reading of between 6.0–6.9% corresponds with 60% developing diabetes within a 10 years follow-up period (20). In this study, similar trends were found for onset age of overweight/obesity which associations were non-significant at a younger-adulthood and mid-adulthood onset age (18), which is similar with our findings. These studies suggest the impact of excessive weight gain in terms of diabetes across the life course, although single comprehensive studies which investigate these changes across a specific nation are few in number.

Excessive weight gain is associated with an increased risk of developing insulin resistance and type 2 diabetes. In obese individuals, adipose tissue releases increased amounts of non-esterified fatty acids, glycerol, hormones, pro-inflammatory cytokines and other factors that are involved in the development of insulin resistance (21). When insulin resistance is accompanied by dysfunction of pancreatic islet beta-cells, failure to control blood glucose levels results in type 2 diabetes. A previous study reported that in addition to initial BMI, obesity onset at younger ages also means cumulative exposure, which is associated with an increased risk of developing type 2 diabetes (22). This supports our finding that an early onset age of excessive weight gain also means a longer cumulative exposure to detrimental factors. Secondly, evidence suggests that earlier excessive weight gain is closely related to genetic predispositions (23, 24). Previous studies have also identified numerous genetic loci and gene variants such as FTO, MC4R, ADAMTS9 and GRB14/COBLL1, which have been found to be associated with overweight/obesity and diabetes (23, 25, 26). Early-onset overweight/obesity participants are likely to be genetically susceptible and carry the above genes which create a higher risk of diabetes. Thirdly, being overweight or obese could interfere with age induced epigenetic changes including DNA methylation, non-coding RNA (ncRNA) and histone modifications. Altered DNA methylation in different tissues (human pancreatic islets, skeletal muscle and adipose tissue) could reduce insulin secretion and increases insulin resistance via differs patterns. Additionally, early onset overweight/obesity may initiate DNA methylation and gene expression in early adulthood and lead to high risk of diabetes (27–32). It is also reported that changes in BMI are accompanied by widespread metabolic changes in early adult, resulting in chronically increased levels of circulating free fatty acids and adipokines, which is again closely associated with diabetes (33–36). Finally, individuals with young-onset overweight/obesity tended to have an unhealthy lifestyle, which likely contributes to the development of diabetes.

In this study, participants with overweight/obesity whose onset age ≥65 years were not observed to be statistically correlate with a higher risk of incident diabetes, compared with normal-weight participants. We supposed that there were some competing risks in older adults who may develop diabetes and obesity simultaneously, reducing the HR of the association. This phenomenon was also observed in other studies (37, 38).

We conducted gender-specific stratification analysis, only observing a positive correlation among women who were overweight or obese and had an onset age of between 45 to 54 years. In overweight or obese women with an onset age <45 years, there was no correlation (aHR, 0.98; 95% CI, 0.47-2.02), which was different from men who had an aHR = 1.52 (95% CI, 1.13-2.04). In a related longitudinal Australian study of women’s health researchers found that obesity onset age negatively correlates with an increased risk of developing diabetes (aHR, 0.87; 95% CI, 0.79-0.96, per 1 year increment) (22). This may have a biological basis because premenopausal women may have a degree of protection from circulating estrogen (39). Another reason could be differences in fat distribution between men and women, where women have a greater proportion of subcutaneous fat and adipose tissue whereas men tend to harbour visceral fat on the abdomin which is a known driver in the progression of disease (40). Therefore, even though these women who became overweight/obese at an onset age <45 years, the risk of developing diabetes mellitus does not necessarily increase. This assertion is supported by sex differences in the prevalences because there are more men with pre-pubescent diabetes, whereas there are more women with postmenapausal diabetes (41). The development of diabetes mellitus after menopause is thought to occur through alterations in insulin secretion, insulin sensitivity, and glucose effectiveness (42).

We observed that the risk of women at onset age between 45 to 54 years was higher than men at same age (aHR, 1.57 *vs* aHR, 1.29), which suggests that the risk of developing diabetes mellitus by weight gain may be much higher in women after menopause. Though a positive correlation was observed in women at onset age higher than 55, such association was not statistically significant. This may be due to the comparatively small sample size of these groups (1,638 at an onset age of between 55 to 64 years and 524 at an onset age higher than 65 years). On the basis of the incidence of 7.26% and 8.20%, the power to find a relationship in these two groups was 0.60 and 0.26 (expected HR = 1.5).

According to previous reports, the risk of obesity may be higher than being overweight in a similar age group (18), but we did not observe that in this study. Hypothetically, this may be due to the comparatively low prevalence of obesity in China when we defined obesity according to World Health Organization (WHO) cutoff (BMI ≥ 30 kg/m^2^). On the other hand, because of the incidence of 6.25% and sample size of 1024, the power to find a relationship in women was only 0.37 (expected HR = 1.5). Therefore, a larger sample is required to explore this further.

Considering the influence of weight loss, we conducted a separate subgroup analysis of overweight or obese category between those who changed to a normal BMI and those maintained in the overweight or obese category. We observed a steady risk reduction in those who changed to a normal BMI, which was consistent with a retrospective cohort study of U.S. adults (HR, 0.33; 95% CI, 0.14-0.76) (43). Surprisingly, the risk reduction effect we observed was steady in all age group. That is to say, weight loss at any age to reduce the risk of developing diabetes.

Apart from diabetes mellitus, researchers have also found weight gain positively correlates to mortality and some cancers, and the younger the onset age of weight gain, the higher the risk (37, 44, 45). A typical study from the United States reported that weight gain at all ages positively relate to mortality, but with stronger associations for weight gain between ages 18 and 35 years and ages 35 and 50 years than between ages 50 and 69 years (44). Another study from the United States showed such relationship between onset age of weight gain and pancreatic cancer (37). The risk change from 1.50 (95% 1.26-1.77) from age 14 to 19 to 1.16 (95% 1.02-1.32) from age 50 to 59, and non-significant from age 60 to 69 (37). Also, age of onset of obesity may, at least in part, affect the prevalence of cardiovascular risk factors in severe obesity (46). Therefore, weight gain over different stages of life from early childhood is implicated in the development of diabetes, specific cancer, cardiovascular diseases and even mortality. Uncertainty remains about whether some life stages are more influential than others.

This was a large prospective study investigating the association between onset age of overweight/obesity and the risk of developing diabetes which use age, rather than study time, as the time scale to optimally account for the observational study design. However, this study also has several limitations. The distribution of gender in our study was imbalanced (men 73.8%) and the characteristics and mechanisms of overweight/obesity and diabetes may be different between men and women. Secondly, our definition of overweight/obesity is based on BMI, which is only an indicator of total body adiposity and could not accurately represent the distribution of adipose tissue or bone density. However, BMI is the most commonly used and practical measure of obesity both in children and adults. There are studies which have proved that BMI is generally consistent with indicators of central adiposity such as waist circumference and waist-to-height ratio (47, 48). Thirdly, the diagnosis of diabetes was based on a single measurement of FBG rather than oral glucose tolerance testing or the measurement of HbA1c, and therefore, the incidence of diabetes might be underestimated. Finally, our regression model included adjustments only for a small number of covariates, which means residual confounders may still exist.

## 5 Conclusion

Our study found that new-onset overweight/obesity correlates with an increased risk of developing diabetes mellitus among Chinese population <65 years. Participants with early onset excessive weight gain or obesity were at a higher risk of having diabetes. The results highlight the importance of preventing the onset of excessive weight gain as we age. Although, further research is needed to link lifestyles, developmental stages, and physiological changes to understand these interactions more clearly.

## Data availability statement

The raw data supporting the conclusions of this article will be made available by the authors, without undue reservation.

## Ethics statement

The studies involving humans were approved by the ethics committee in Kailuan General Hospital. The studies were conducted in accordance with the local legislation and institutional requirements. Written informed consent for participation in this study was provided by the participants’ legal guardians/next of kin.

## Author contributions

WF: Formal Analysis, Software, Writing – original draft. XY: Formal Analysis, Software, Writing – original draft. WL: Data curation, Writing – review & editing. SS: Writing – review & editing. GC: Data curation, Writing – review & editing. ZFC: Data curation, Writing – review & editing. ZH: Data curation, Writing – review & editing. XW: Data curation, Writing – review & editing. WW: Data curation, Writing – review & editing. ZCC: Data curation, Writing – review & editing. YL: Funding acquisition, Methodology, Writing – review & editing. SW: Conceptualization, Project administration, Writing – review & editing. YC: Conceptualization, Project administration, Writing – review & editing.
