# Supplementary material for: Excessive weight gain onset-age and risk of developing diabetes mellitus: a large, prospective Chinese cohort study
Source: Front Endocrinol (Lausanne). 2023 Nov 27;14:1281203. doi: 10.3389/fendo.2023.1281203 (PMC10711082; doi:10.3389/fendo.2023.1281203)
Supplement: Supplementary file 1 [file DataSheet_1.docx]

Supplementary Material

# Supplementary Data

Questionnaire of diabetes history and treatments (Translation Form)

# Supplementary Figures and Tables

**Figure S1.** Participant inclusion process and Schedule of matching and study visits. (A) Participant inclusion process through the study. n, number of individuals in study as per protocol. (B) Schedule of matching and study visits. Red star means new-onset overweight/obese subjects. Green circle means matched normal-weight subjects.

**Table S1.** Missing covariate rates.

**Questionnaire for diabetes mellitus history and treatments (Translated version)**

Q1. Have you been diagnosed with diabetes mellitus by a third class hospital before?

(1) Yes; (2) No

Q2. If yes in Q1, did you receive any treatment？

(1) Yes; (2) No

Q3. If yes in Q2, which of following treatments were you prescribed?

(1) Insulin; (2) Sulfonylurea; (3) Biguanide; (4) Glucoside inhibitors; (5) Thiazolidinediones; (6) Non-insulin pro-secretory agents; (7) Traditional Chinese medicine and other.


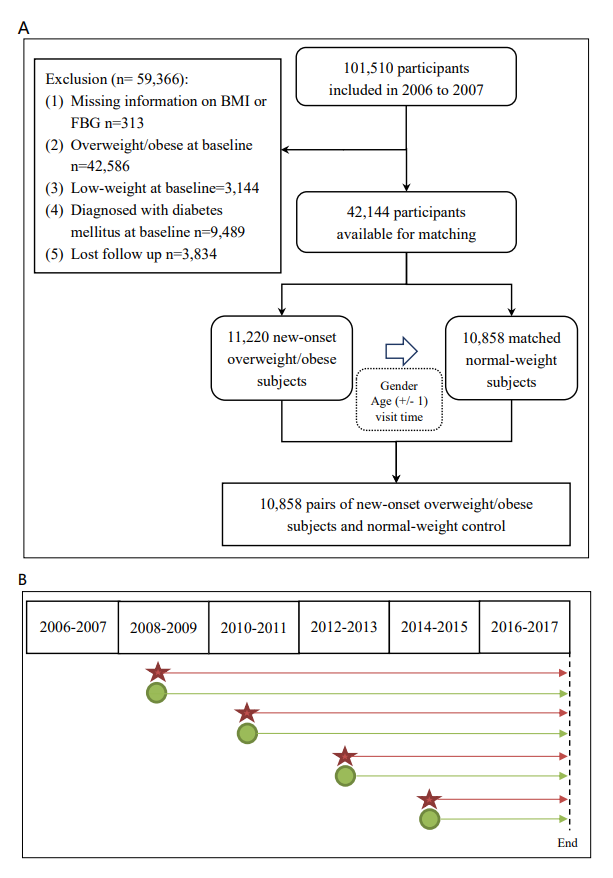


**Figure S1. Participant inclusion process and Schedule of matching and study visits.** (A) Participant inclusion process through the study. n, number of individuals in study as per protocol. (B) Schedule of matching and study visits. Red star means new-onset overweight/obese subjects. Green circle means matched normal-weight subjects

**Supplementary Table 1**. Missing covariate rates

| **Variables** | **Total** | **Missing** | **Rate (%)** |
| --- | --- | --- | --- |
| SBP | 21,716 | 186 | 0.86 |
| DBP | 21,716 | 185 | 0.85 |
| HDL-C | 21,716 | 191 | 0.88 |
| LDL-C | 21,716 | 217 | 0.99 |
| Smoking | 21,716 | 0 | 0 |
| Drinking | 21,716 | 1 | 0.05 |
| Physical exercise | 21,716 | 0 | 0 |
| Family history of diabetes | 21,716 | 0 | 0 |
| Education | 21,716 | 533 | 2.45 |

Notes: DBP = diastolic blood pressure; HDL-C = high-density lipoprotein-cholesterol; LDL-C = low-density lipoprotein-cholesterol; SBP = Systolic blood pressure.
